# Monitoring Construction Workers’ Mental Workload Due to Heat Exposure Using Heart Rate Variability and Eye Movement: A Study on Pipe Workers

**DOI:** 10.3390/s25082377

**Published:** 2025-04-09

**Authors:** Shiyi He, Dongsheng Qi, Enkai Guo, Liyun Wang, Yewei Ouyang, Lan Zheng

**Affiliations:** 1 Key Laboratory of Physical Fitness and Exercise Rehabilitation of Hunan Province, Hunan Normal University, Changsha 410006, China; heshiyi@hunnu.edu.cn (S.H.); dongshengq@126.com (D.Q.); guoenkai18@126.com (E.G.); 202130175032@hunnu.edu.cn (L.W.); 2 College of Civil and Transportation Engineering, Shenzhen University, Shenzhen 518060, China

**Keywords:** mental workload, HRV, eye movement, heat exposure, pipe workers

## Abstract

Monitoring the mental workload of construction workers is effective in detecting risky subjects because cognitive overload may threaten their safety. This study aimed to measure workers’ mental workload caused by heat exposure using heart rate variability (HRV) and eye movement features. Inexperienced pipe workers (*n* = 30) were invited to perform an installation task in a normothermic (26 °C, 50% RH) and a hyperthermic (33 °C, 50% RH) condition. Their HRV and eye movement features were recorded as the inputs of training models classifying mental workload between the two thermal conditions, using supervised machine learning algorithms, including Support Vector Machines (SVM), KNearest Neighbor (KNN), Linear Discriminant Analysis (LDA), and Random Forest (RF). The results show that applying eight HRV features through the KNN algorithm could obtain the highest classification accuracy of 90.00% (Recall = 0.933, Precision = 0.875, F1 = 0.903, AUC = 0.887). This study could provide a new perspective for monitoring the mental workload of construction workers, and it could also provide a feasible approach for the construction industry to monitor workers’ mental workload in hot conditions.

## 1. Introduction

Construction workers are in a high-risk occupational environment because construction sites are complex and involve many dangerous tasks. It is essential for workers to keep an excellent cognitive state to ensure their safety in workplaces [1]. Mental workload is a typical cognitive state that is closely associated with workers’ safety performance. It refers to the proportion of the human’s mental capabilities occupied when performing a given task [2]. When demands on a task surpass an individual’s limited cognitive resources, the mental workload can become overloaded, leading to a rapid decline in performance [2]. Mental overload can hinder subjects’ performance and safety by raising error rates, inducing fatigue, reducing motivation, prolonging reaction times, and causing ignoring of crucial information [3]. It is demonstrated that mental load negatively affected situation awareness, which determines workers’ risk perception and comprehension and ultimately impacts workers’ safe behavior [4]. In addition, when workers perceive a high level of mental workload on job sites (i.e., most of their attentional resources are occupied), they may become unintentionally blind, which can reduce their perceptual abilities and make them more vulnerable to danger [5].

Construction workers’ mental workload may be induced by task’s properties, the working environment, and the human operator’s characteristics in construction workplaces [3]. Heat exposure is among the environmental factors. Mental workload may be increased by heat exposure because hyperthermia is an ongoing factor during the primary task and may have used similar cerebral resources as the primary task when working in hot conditions [6]. The number of elements that need attention during mental activities is the major contributor to cognitive load [7]. Compensatory activity is unnecessary within a subject’s comfort zone, but additional attentional resources are crucial to sustain performance at the same level outside the comfort zone, such as in hyperthermic situations [8]. This additional mental effort will generate an extra cognitive load for subjects. However, hot working environments are frequent in the workplaces of construction workers, especially for those performing work outdoors without shading [9]. What is worse, the temperature that construction workers experience in workplaces can be much higher than the air temperature. The air temperature of 57 °C inside a worker’s safety helmet was recorded in August of Hong Kong when the environmental temperature was 33 °C [10]. Given these conditions, it becomes critical to monitor workers’ mental workload in heat-exposed environments to enhance occupational safety.

However, current research in the construction industry has not specifically addressed the mental workload induced by heat exposure. Most existing studies have concentrated on task-related factors, such as the complexity of tasks, safety information, and individual traits like cognitive styles [11]. Despite this, there is a clear gap in understanding the impact of environmental stressors, particularly heat, on workers’ cognitive load in construction settings.

Methodologies for assessing an individual’s mental burden consist of subjective, behavioral, and physiological measures [12]. Subjective measures typically involve self-assessment scales or interviews. The NASA Task Load Index (NASA-TLX) is a typical scale used to measure mental workload. It comprises six dimensions that reflect mental workload: mental demands, physical demands, time demands, performance, effort, and frustration levels [13]. Depending on subjects’ self-reports is constrained due to the disparity between a person’s objective status and their subjective perception of it. Additionally, obtaining a worker’s self-assessment is cumbersome and unfeasible in construction settings. It is not feasible to measure a person a lot, let alone constantly. When using subjects’ primary- and secondary-task performances to assess mental workload, the performance-based data can be misleading as different levels of mental workload can result in similar performance levels [14]. In contrast, physiological measures have advantages over the two ways. Physiological metrics are more accurate and reliable since physiological reactions cannot be manipulated at will [15]. Physiological measures can also function continuously, e.g., online fatigue measuring [16]. The physiological measurement method shows promise in monitoring construction workers’ mental workload in workplaces with minimal disruption to building activity [17].

Previous research in the construction field has quantified the negative effects of mental workload (MW) on workers using physiological measures. These studies have primarily focused on EEG. For instance, the study of [18] examined EEG indicators that could reveal workers’ mental workload caused by various construction task demands, while the study of [5] developed a measurement approach to evaluate hazards through neural time–frequency analysis of EEG features. Additionally, the study of [19] utilized EEG to predict subjects’ mental workload during Augmented Reality head-mounted display tasks for construction assembly. However, using EEG for real-time monitoring on construction sites has significant limitations. Subjects must maintain a state of stability and tranquility, while the required equipment tends to be costly and intricate. EEG signals are also susceptible to interference from surrounding noise, and the monitoring process involves using intrusive headsets, which can be invasive. As a result, the practical application of EEG devices on construction sites remains constrained.

Electrocardiograph (ECG) readings, which are easier to obtain than neural signals in construction sites, could be a viable method for assessing workers’ mental workload. ECG signals reveal the heart’s rhythm and electrical activity by placing sensors on the skin to detect the electrical impulses generated by the heart with each beat. Signals can be gathered via wristbands, armbands, or chest straps [20], enhancing the feasibility of ECG sensors in real-life conditions. Heart rate variability (HRV) is a significant characteristic of ECG data that shows potential in assessing mental workload. It is an important metric derived from ECG signals, indicating the variation in the time durations between consecutive heartbeats. The phenomenon is produced by interactions between the heart and brain, as well as dynamic non-linear processes of the autonomic nervous system (ANS) [21]. Mental workload directly affects ANS [22]. Since the ANS is also responsible for the control of the cardiovascular system, the mental workload compromises the normal regulation of the heart rate (HR) and electrophysiology of the cardiac cells [23]. Thus, the electrocardiogram is considered a suitable physiological signal for measuring the impact of mental workload on the human body [24,25]. HRV has been shown to quickly respond to changes in momentary workload in previous research [24]. For example, a study [25] collected 20 HRV parameters from ECG data to classify mental effort in people using a dual-arm robot, with an average accuracy of 98.77%. The research [26] found that rising traffic congestion led to a higher mental workload for operators, which in turn had a notable impact on HRV features. HRV has been widely used to investigate autonomic nervous system activity in various contexts, such as surgery, car driving, and robot systems [27].

Eye-tracking, in addition to ECG, can be employed to assess mental overload by capturing the subject’s eye movements and gaze orientations. Cortical activity due to mental workload would lead to a small nervous response that would be translated into variations in eye movements and small dilations of the pupil [28]. Several eye-movement indicators, including fixation-based, saccade-based, pupil dilation, and blink rate, are demonstrated to be associated with changes in mental workload [29]. The majority of research analyzing blink rate (71%), pupil diameter (79%), and fixation length (73%) found a statistically significant difference in distinguishing activities with different mental effort levels [29]. More importantly, eye movement data can be collected from workers at construction sites, even under hot conditions. The acquisition of eye movement data is facilitated by wearable devices, such as the Tobii Glasses 3, which allow for the tracking of workers’ eye movements in dynamic environments. This data collection method is negligible to disrupt the worker’s building activities, because eye movements remain unaffected by heat exposure. The processing of eye movement data is also straightforward, enabling real-time computing. Therefore, this study will also explore the potential of eye movement features for predicting the mental workload.

Therefore, this study intends to develop predictive models based on workers’ heart rate variability (HRV) and eye movement to estimate the mental workload induced by heat exposure to fill the gap. This would be a good approach to apply at job sites. The approach can provide objective data, function continuously, and with minimal interference to construction activities. It is also feasible to acquire physiological data in construction workplaces. The outcomes would offer a method for assessing employees’ cognitive workload induced by heat exposure at work locations. It could also provide more insights into the connections between workers’ physiological characteristics and mental workload.

## 2. Materials and Methods

**Figure 1:**
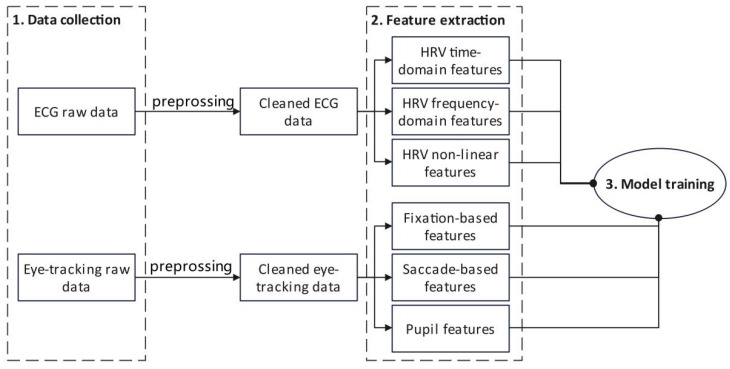
Research framework.

This study utilized laboratory trials and machine learning approaches to create prediction models for assessing workers’ mental workload. As shown in Figure 1, at first, raw data on ECG signals and eye movement were acquired when pipe workers performed the task in the lab under two different heat settings. After preprocessing to remove noise components, HRV features and eye-tracking metrics related to mental workload were computed. And then, machine learning methods were applied to develop prediction models indicating workers’ mental workload while the two types of data were used as inputs for training. The details are clarified in the following sections.

### 2.1. Experimental Design

#### 2.1.1. Subjects and Apparatus

A total of 30 male pipe construction workers volunteered for this study. The participants were screened for visual health and had uncorrected or corrected visual. Individuals with cardiovascular diseases, smokers, and those taking medications that affect HRV were excluded from the study. They were aged 22 to 31 (M = 25.9 years; SD = 2.7 years), with less than three years of experience in pipe installation. Specifically, among the thirty participants, nineteen had less than one year of experience, seven had between 1~1.5 years of experience, and four had 2~3 years of experience. According to China’s “Piping Worker Occupational Skill Standards” [30], there is no explicit definition of the number of years of experience that constitutes a novice. However, the standards categorize professional qualifications, specifying that workers who have held an intermediate-level certification can only apply for a higher-level qualification after working in the field for at least 3 years. Additionally, after consulting experts, it was determined that workers with less than 3 years of experience are typically in the skill development phase and have not yet reached the level of proficiency. Therefore, those with less than 3 years of experience were considered as novices. Furthermore, most of the participants in the sample had less than 1 year of experience. Pipe workers were chosen because they are required to read construction drawings and lay out the pipes of various professionals based on the designed pipe elevations and alignments, which involves the use of more cerebral resources to complete the task compared to other types of construction trades. The inexperienced workers were invited because they would be more sensitive to the task difficulty and were more easily impacted by mental workload due to a lack of experience. Participants were instructed to adhere to their regular sleep patterns, refrain from intense physical exertion, and abstain from consuming caffeine and nicotine for the 24 h prior to the experiment. Before the experiment started, the participants were also asked if they felt any discomfort, to ensure that they were in a good mental state and free from any discomfort during the experiment. The study was authorized by the Ethical Research Committee of Hunan Normal University. Additionally, each participant provided informed written consent.

ECG signals were collected using the Custo Guard (Custo Med GmbH, Ottobrunn, Germany). One electrode of the holter was placed in the one lead, which refers to the fourth intercostal space just to the right of the sternum. Data were recorded using the ECG sensor at a 512 Hz frequency rate. The eye camera of the eye-tracker captured and recorded subjects’ real-time eye movement. A remote eye-tracker (Tobii Glass 3) was used to record eye movement data at a frequency of 100 Hz.

#### 2.1.2. Experimental Conditions

There are two thermal conditions: normothermic (NT) and hyperthermic (HT). The normothermic condition included an ambient temperature of 26 °C and a 50% relative humidity (26 °C, 50% RH); 26 °C is a comfortable temperature for the human body. To accommodate this range, the chamber’s air temperature was set to 26 °C, with a humidity level of 50%. The hyperthermic situation is characterized by a hot and humid environment with an average air temperature of 33 °C and a relative humidity of 70% (33 °C, 70% RH). The temperature of 33 °C was chosen because employers are required to pay “high-temperature subsidies” to employees above this temperature level in China [31]. The 70% relative humidity was chosen because it is generally regarded as the upper limit for thermal comfort in hot and humid settings [32]. In addition, the effect of humidity on human response would not be significant until relative humidity reached 70% [32]. The experiment was conducted in a 2.4 × 4.0 × 2.8 m^3^ climate chamber. Besides controlling temperature and humidity, other environmental variables were also managed. Lighting conditions were stabilized using uniform indoor illumination, acoustic disturbances were minimized by conducting all trials in an isolated single-room setting, and CO_2_ concentrations were maintained within standardized thresholds via a mechanically regulated ventilation system.

#### 2.1.3. Experimental Process

**Figure 2:**
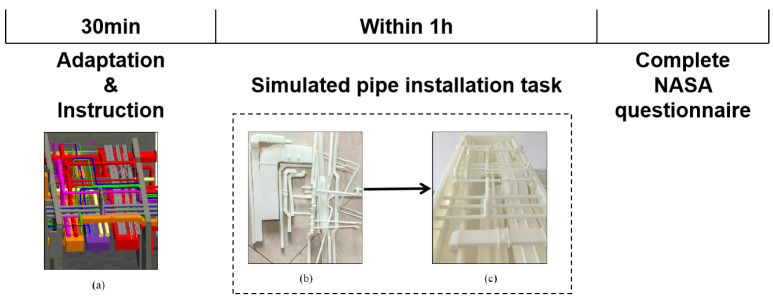
Experimental process. (**a**) 3D digital models (built in Autodesk Revit); (**b**) 3D printed components; (**c**) 3D printed models (being assembled).

The experimental procedure is shown in Figure 2. First, the participants adapted to the laboratory environment for half an hour, during which the experimenter explains the experimental tasks. The participants were required to complete the experiment within one hour. Specifically, the subjects were required to perform a simulated pipe installation operation. The piping systems were extracted from three rooms in a hospital project (as shown in Figure 2). Using a 3D printing technique, the models of piping systems and building structures were printed into three dimensional (3D) models (as shown in Figure 2) at a scale of 1:30 using the 3D printing technique. During the experiment, the workers were required to install the pipes in the space (as shown in Figure 2) according to the provided two-dimensional drawings. Their task performance was measured by completion time, as the model can only be correctly assembled when all components are correctly placed. If the model is built incorrectly, some components will not be able to be integrated into the model. After completing the experiment, participants were required to complete the NASA questionnaire. Subjective responses about mental workload were gathered to determine if heat exposure caused an increase in workers’ mental workload. Participants completed the NASA-TLX questionnaire [13] following the task to assess their mental effort while executing the task. The NASA-TLX questionnaire, a standard questionnaire for subjects self-reporting their mental workload [13]. The scale comprises six components assessing mental demands, physical demands, temporal demands, performance, effort, and frustration level. Each item can be rated on a 10-point scale ranging from 1 (very low) to 10 (extremely high). The total workload estimate was calculated by averaging all six elements.

Moreover, to eliminate the effects of task sequence variations and individual differences, we implemented a randomized crossover design. Specifically, all participants were randomly divided into two groups: Group 1 completed the task under NT first, followed by HT. Group 2 performed the tasks in the reverse order. The two experimental phases were separated by a one-month interval to minimize carryover effects.

### 2.2. Data Collection and Pre-Processing

ECG data collected during the task were utilized as input for predictive algorithms. The raw ECG data were processed using Kubios HRV Premium version 3.5.0 [33] to eliminate noise. Kubios incorporates an adaptive QRS detection method together with automatic and threshold-based artifact reduction techniques to eliminate noisy regions in the raw signals. The R-wave time points were identified using the QRS detection algorithm known as the Pan–Tompkins algorithm [34]. The QRS detection algorithm utilized pre-processing techniques to eliminate noise, such as band-pass filtering of the ECG to reduce power line noise, baseline wander, and other noise components. Additionally, the data samples were squared to emphasize peaks, and moving average filtering was applied to smooth nearby peaks [33]. Automatic noise detection was used to identify noise regions in the raw ECG data.

Tobii Pro Lab software (V.1.181, Tobiitech, Stockholm, Sweden) was applied to record the eye movement data and compute features. A Tobii Velocity-Threshold Identification Gaze Filter fixation filter (Tobii I-VT) was adopted to categorize eye movements according to the speed of the eye’s directional changes. An Attention Filter, designed for processing eye-tracking data from glasses recordings, was chosen with a velocity threshold of 100 degrees/second. The mean positions where the left and right eyes were looking were utilized as the units of analysis.

### 2.3. HRV Features Computation

This study selected 15 HRV features listed in Table 1 that might have potential in predicting the mental workload [13,26,35,36], including 4 time domain features, 8 in the frequency domain, and 3 non-linear features.

The mental workload directly affects the autonomic nervous system (ANS) [23]. The ANS consists of the sympathetic nervous system (SNS) and parasympathetic nervous system (PNS). Time-domain characteristics (mRR, SDNN, RMSSD, and PNN50) were estimated to predict mental workload, reflecting changes in the sympathetic nervous system (SNS) and parasympathetic nervous system (PNS). mRR represents the mean time interval between two successive normal R peaks in an ECG signal. Both SNS and PNS activities influence SDNN. The RMSSD measures the variability in heart rate from beat to beat and is the major time-domain metric for assessing the vagally driven alterations observed in HRV [21]. The pNN50 is strongly associated with PNS activity [21].

Frequency-domain features can comprehensively indicate the ANS activity. The features (VLF, LF, HF, TP, LF/HF, nLF, nHF, pVLF, pLF, pHF) were calculated to provide additional insights into the characteristics of oscillatory components in heart rate dynamics. The analysis focused on four primary spectral components: VLF, LF, HF, and TP (a measure of overall autonomic activity). The activity of PNS activity may influence VLF power, LF power may be produced by both the PNS and SNS, and the HF band reflects PNS activity [21]. Among varied frequency-domain HRV measures, LF/HF ratio was the most widely used measure, followed by high frequency, low frequency, and mid-frequency [29]. Except for the absolute power (ms^2^), LF and HF were also measured in normalized units (n.u.), i.e., nLF and nHF, and their percentages were calculated (%), i.e., pVLF, pLF, and pHF. Furthermore, the LF/HF ratio, representing the equilibrium between parasympathetic and sympathetic activity [37], was also calculated.

In addition, non-linear features (SD1/SD2, ApEn, SampEn) were computed. The ratio between SD1 and SD2 could reflect sympathetic activation [29]. Entropy quantifies the intricacy or randomness of the time sequence. A lower entropy value signifies a lower level of data complexity. Approximate entropy (ApEn) and sample entropy (SampEn) are metrics that quantify irregularity or complexity in the series.

### 2.4. Eye Tracking Features Computation

Fixation-based, saccade-based, and pupil dilation indicators linked to variations in mental workload were selected [29], as shown in Table 2. Fixations are those states when an individual’s eyes pause their scanning of the environment, allowing the foveal vision to focus on specific details for the visual system to process. Fixation is generally associated with attention, visual processing, and information absorption [38]. The total fixation duration refers to the cumulative time spent fixating during the entire task. The average fixation duration is calculated by dividing the total duration of fixation periods by the total number of fixations. The fixation count is the whole number of fixations made throughout the entire job. Extended fixation duration and a higher number of fixations have been demonstrated to indicate an increased mental workload [29].

Saccades are rapid eye movements between fixations. Saccades are essential for bringing areas outside the focal area into focus or our attention; thus, they are central to navigating the visual world [38]. Saccade count is the number of saccade events during the task, and the more the saccades, the higher the mental workload might be [36]. The average velocity of saccades refers to the average angular velocity of all saccade events. An increase in saccade velocity was found to indicate a higher task difficulty [39]. The average amplitude of saccades refers to the average length from the center of the fixation points before the saccade to the center of the first fixation point after the saccade. Longer saccades were demonstrated to correspond to a higher load [28]

Studies have demonstrated that pupil diameter generally increases with an increase in mental load [30]. Mean pupil diameter is utilized as an indicator of tonic dilation, with mean pupil diameter values increasing with an increase in mental load [40]. Nevertheless, when considering the mean value of pupil diameter over the entire time series, it fails to capture the phasic fluctuations in pupil diameter, that the high and low fluctuations may cancel each other out [41]. The standard deviation of pupil diameter has therefore also been selected to indicate phasic fluctuations, with a higher standard deviation of pupil diameter suggesting a higher fluctuation in workload levels [41].

### 2.5. Prediction Model Development

Supervised learning algorithms are applied to create prediction models because to their proven ability to efficiently, accurately, and reliably detect cognitive states by analyzing specific physiological characteristics [42]. The dataset of HRV and eye-tracking features were labeled at first. It is expected that there will be a significant difference in the workers’ mental workload when performing the task under the two thermal conditions. The workers are expected to suffer low mental workload under the normothermic condition and to experience a higher level of load in the hyperthermic condition. To validate the accuracy of the data labeling, workers’ subjective feedback of the mental workload of the task was collected.

Regarding developing prediction models, feature selection was first conducted, which could make the model simpler and easier to interpret, avoid overfitting, and reduce the computational cost of training a model. Statistical analysis was applied to identify the most relevant features. A paired-T test was for feature selection in the training process. The importance of the features is ranked according to the absolute values of t. The larger the absolute value of t, the more this feature distinguishes between the two types of samples. All the features will be used for training at first; then, one feature would be removed at a time to retrain the model according to the absolute value of the t-value. Features with a smaller absolute t-value would be removed first because they contribute less to distinguishing the two types of samples. The reason for using the absolute value of t to rank features instead of the significant p-values is that while features with significant p-values can distinguish between the two types of samples, relying solely on features with significant p-values may cause some useful features to be overlooked, thereby reducing the model’s performance. After feature selection, normalization was performed using the Min–Max Scaling to translate data into the range [0, 1] to eliminate the influence of the scale among features.

Cross-validation was employed to enhance the model’s generalization and prevent overfitting. The leave-one-out cross-validation (LOO) strategy is used to separate the training and test datasets. LOO partitions the data into one subject as the test dataset and the others as the training dataset, allowing it to utilize each subject’s sample fully to minimize subject bias resulting from individual differences [43]. LOO can be especially useful for small sample size, and it can help to prevent overfitting by providing an estimate of the model’s performance on new data [44]. Grid search was also adopted for hyperparameter optimization to systematically cut down the search range of hyperparameters and streamline the process of finding the best hyperparameters.

Four supervised learning algorithms chosen for model training are Support Vector Machines (SVM), KNearest Neighbor (KNN), Linear Discriminant Analysis (LDA), and Random Forest (RF). SVM constructs hyperplanes to distinguish data points in a binary classification problem. SVM is appropriate for high-dimensional data with non-Gaussian distribution, a common trait of many psychophysiological datasets [45]. KNN utilizes the complete database to make predictions by assessing similarity in the instance space [46]. KNN is resilient to noisy data and has a little computing cost, making it an appealing choice for applications in industry [42]. LDA establishes separating hyperplanes by identifying the direction in feature space with the largest inner-means distance and the smallest variance [47]. The method is straightforward and has been effectively utilized in numerous Brain Computer Interface (BCI) applications [48]. The RF classifier provides dependable classifications by utilizing predictions from a group of decision trees [49], which has been proven to be resistant to noise in ECG data [50]. This study examined linear and RBF kernels for SVM, with hyperparameters C and γ defined within the range of [2–5, 25]. The hyperparameter K for KNN is defined within the range of 1 to 25. For RF, Ntress ranges from 10 to 1000 trees in increments of 100, and Mtry ranges from 2 to the maximum number of features available.

Finally, the algorithms’ classification ability was assessed based on mean accuracy, recall, precision, F1 score, and the area under the receiver-operating characteristic (ROC) curve (AUC). This study categorizes samples in hyperthermic conditions as positive and samples in normothermic conditions as negative. The recall is determined by the number of true positive samples divided by the total number of positive samples. The recall measures the model’s capacity to detect positive samples. Increased recall results in the detection of more positive samples. The recall value is examined because the prediction models aim to detect workers experiencing a higher mental workload. Precision is defined as the ratio of correctly classified positive samples to the total number of classified positive samples. Precision allows us to assess the machine learning model’s reliability in classifying positive examples. F1 score provides a tradeoff between precision and recall. It is the harmonic mean of precision and recall. The AUC is a reliable metric for evaluating and comparing the effectiveness of various characteristics or classification algorithms. A higher AUC value indicates a better classification performance. The proposed classification algorithms and the classification evaluation were implemented in Python 3.7.

## 3. Results

### 3.1. Task Performance and NASA-TLX

**Figure 3:**
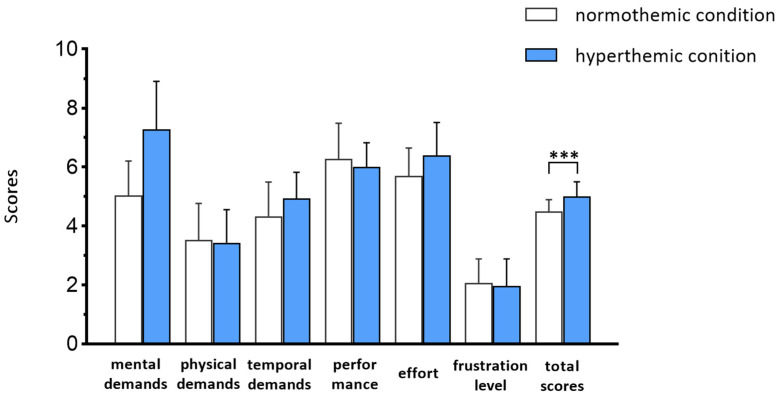
Results of mental workload by subjective reports. Note: ***, vs. normothemic condition, *p* < 0.001.

The results of the completion time under the two conditions are: normal temperature (Mean = 46 min, SD = 1.3 min) and high temperature (Mean = 47 min, SD = 1.22 min). There are no significant difference (*p* = 0.43). The results show that the average completion time was slightly longer under high temperature, which supported the idea that workers suffered higher mental workload, and this is consistent with the participants reporting higher time demands under high-temperature conditions (as shown in Figure 3).

The results of the reported mental workload are shown in Figure 3. The mean NASA score of the normothermic and hyperthermic conditions are, respectively, 4.49 and 5.00. The heat exposure significantly increased workers’ mental workload (*p* < 0.001).

### 3.2. Statistical Results of HRV and Eye Movement Features

The paired T-test was applied to compare the differences in HRV and eye movement features between the two conditions, with results shown in Table 3 and Table 4. The ascending order according to the absolute values of t of HRV features is SampEn, VLF, SD2/SD1, SDRR, pVLF, TP, nHF, nLF, ApEn, HF, pHF, mRR, RMSSD, pLF, LF, PNN50, and LF/HF. The SampEn, SD2/SD1, and VLF showed significant differences. Among the time-domain features, the mean value of mRR decreased, and the average SDRR, RMSSD, and PNN50 increased. Regarding frequency-domain features, the mean values of VLF, HF, TP, pVLF, pLF, and nHF decreased, while the average LF, pHF, LF/HF, and nLF increased. The three non-linear increased on average.

As shown in Table 4, the ascending order according to the absolute values of t of eye movement features is TFC, SC, MFD, TFD, SPD, MSV, MSA, and MPD. There were significant differences in all of the fixation-based features and the saccade-based feature (SC). The total fixation duration, total fixation count, and saccade count significantly increased. The mean values of saccade velocity, saccade amplitude, mean pupil diameter, and standard deviation of the pupil diameter increased. When HRV and eye movement features are combined, the ascending order according to the absolute values of t is TFC, SC, MFD, SampEn, TFD, VLF, SD2/SD1, SDRR, pVLF, TP, nHF, nLF, SPD, ApEn, HF, pHF, mRR, RMSSD, MSV, MSA, MPD, pLF, LF, PNN50, and LF/HF.

### 3.3. Model Evaluation

**Figure 4:**
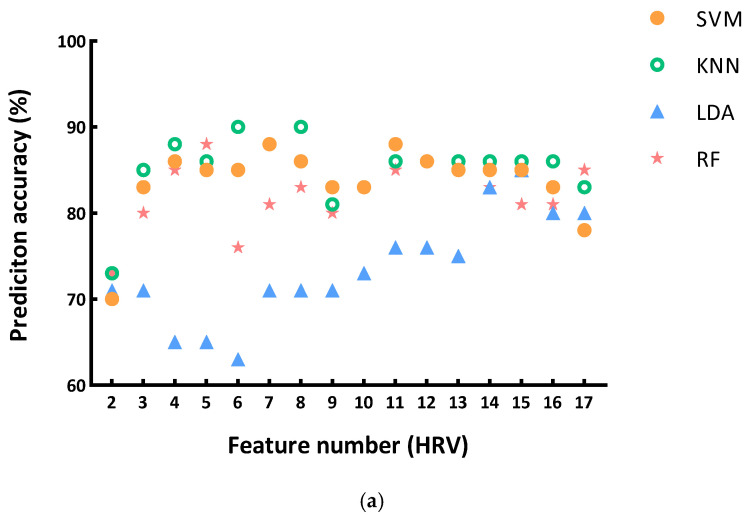

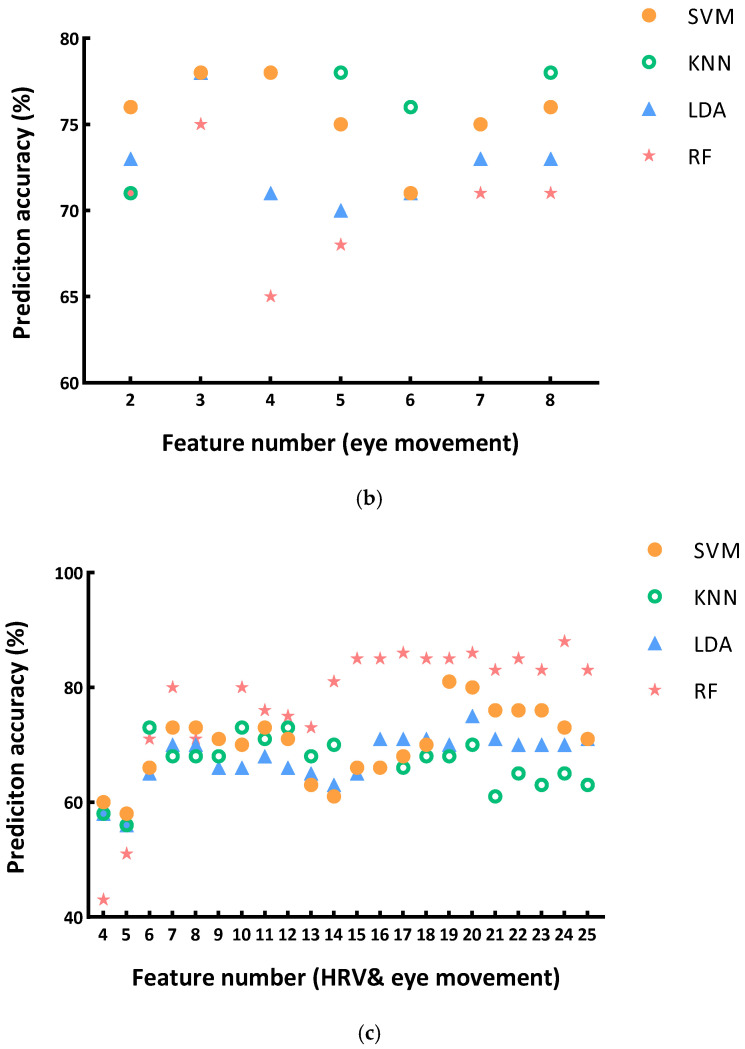
Results of the classification accuracy: (**a**) models using HRV features; (**b**) models using eye movement features; (**c**) models using HRV and eye movement features.

The prediction accuracy results are summarized in Figure 4, which shows the accuracy of the four applied algorithms (SVM, KNN, LDA, and RF) when various numbers of features are selected. Figure 4a shows that applying eight or six HRV features could achieve the highest accuracy of 90.00% using the KNN algorithm (k = 3). Figure 4b shows that there are several eye movement feature combinations which could achieve the highest accuracy of 78.33%. The KNN algorithm performed the best when there were eight features (k = 14), five features (k = 7), four features (k = 7), and three features (k = 8). The SVM algorithm showed the best accuracy when applying four or three features. The LDA algorithm also obtained the highest accuracy when using three features. Figure 4c shows that when using twenty-four HRV and eye movement features, the RF algorithm obtained the highest accuracy of 88.33%.

**Figure 5:**
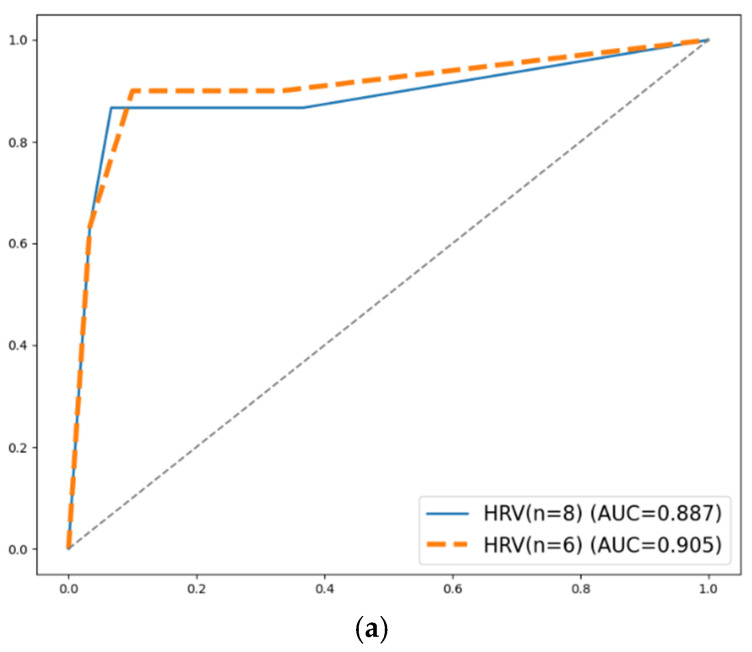

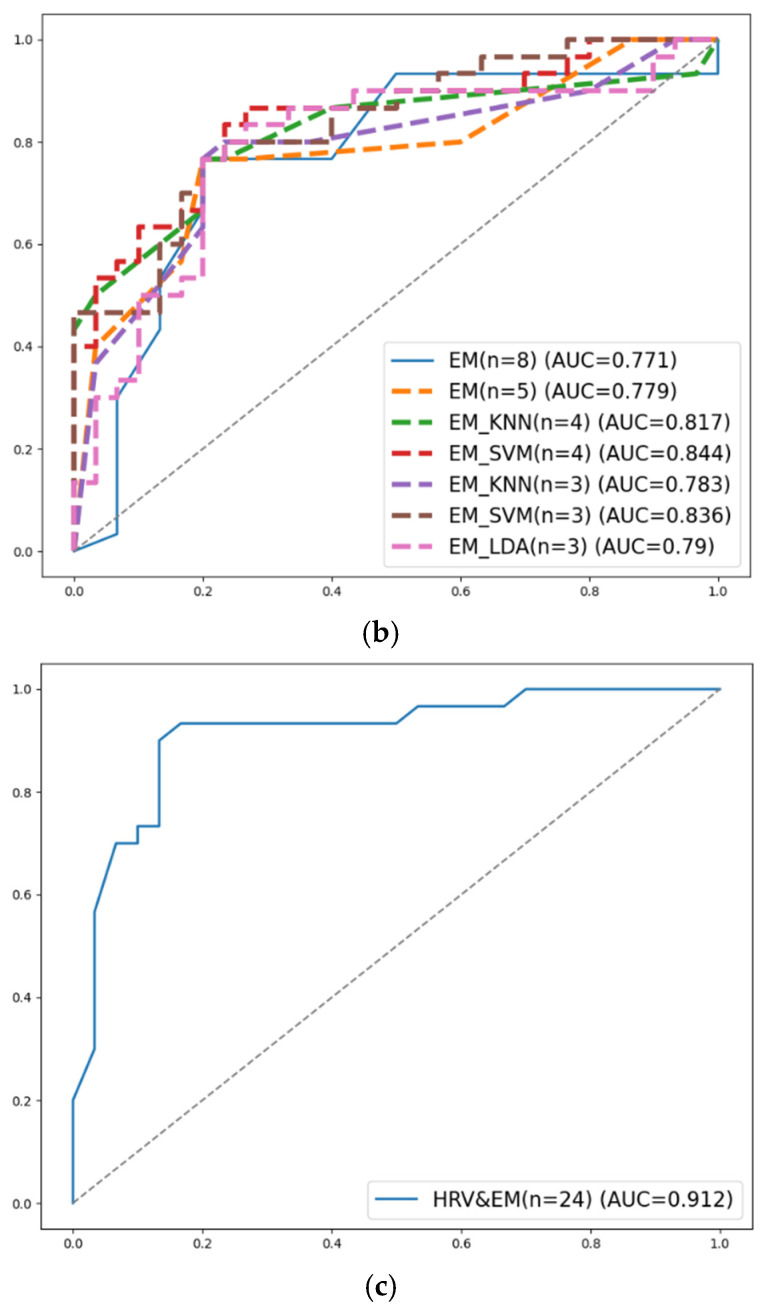
ROC curves of prediction models with the highest prediction accuracy: (**a**) models using HRV features; (**b**) models using eye movement features; (**c**) models using HRV and eye movement features.

**Figure 6:**
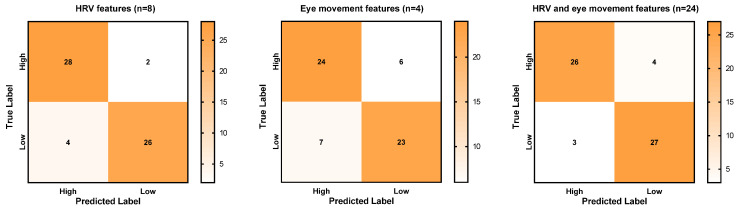
Confusion matrix of the prediction models with better evaluation performance.

Table 5 shows the results of evaluation metrics of these prediction models with the highest accuracy, as shown in Figure 4, and their ROC curves are shown in Figure 5. Regarding models using HRV features, the model applying eight features was slightly poorer than the one using six features in precision, F1, and AUC, but it had a larger recall value (0.933). The model aims to detect workers’ mental workload, so the recall value, which demonstrates the ability of the model to detect mentally overloaded samples, is relatively more important than other evaluation metrics. It is hence suggested to adopt the model applying eight HRV features, with its confusion matrix shown in Figure 6. The applied eight HRV features are SampEn, VLF, SD2/SD1, SDRR, pVLF, TP, nHF, and nLF.

Regarding the seven models using eye movement features, they had the same values of recall, precision, and F1. The evaluation performance showed a different AUC value. The model with four features using the SVM algorithm had the highest AUC value (0.844), which performed the best among the seven models (with confusion matrix shown in Figure 6). The applied four eye movement features are total fixation duration (TFD), average fixation duration (MFD), total fixation count (TFC), and saccade count (SC).

In terms of using both HRV and eye movement features, the model performed well in recall, precision, and F1, and it also had an AUC of over 0.900. Among the total 25 features, only the LF/HF was not applied. Its confusion matrix is shown in Figure 6.

## 4. Discussion

### 4.1. Physiological Features and Mental Workload

This study screened for features that could better differentiate the mental workload levels by statistical tests. Among the three types of HRV features, the mental workload is less sensitive to the time domain features compared to the frequency domain and non-linear features. None of the time-domain indicators had significant differences, and they had a relatively small t-value. Time-domain indices may not offer extensive information on sympathetic control, whereas certain frequency bands are believed to indicate sympathetic and/or parasympathetic regulation, providing more detailed insights into the autonomic nervous system [27]. As for non-linear metrics, they are of potential to reliably capture the complexity of HRV [51].

The collected frequency-domain and non-linear HRV features HRV are reasonable. Human’s stress response occurs when their mental workload increases, with the sympathetic nervous system (SNS) being activated and the parasympathetic nervous system (PNS) inhibited [25]. The LF band (0.04~0.15 HZ) is regulated by the sympathetic nervous system, and the HF (0.15~0.4 HZ) band is regulated by the parasympathetic nervous system [21]. Therefore, there would be an increase in LF and a decrease in HF. Although there were no significant differences in the HF and LF, their changes in average values are in line with the pattern. The results of non-linear features are consistent with the previous findings where the SD2/SD1, ApEn, and SampEn increased when suffering mental workload [52].

In terms of the three types of eye movement features, the mental workload is more sensitive to the fixation-based features than the saccade-based and pupil diameter features in our results. The three fixation-based metrics all showed significant impacts on mental workload, with large t values. Fixations indicate human’s information processing information absorption [38]. Hence, subjects with larger cognitive load are expected to show longer fixation duration or more fixation numbers [53]. Our results correspond to the expectation, where the workers’ total fixation duration and fixation counts significantly increased when suffering a higher level of mental workload in the hot condition. Saccades are rapid eye movements between fixations. It was demonstrated that the saccade numbers, saccade velocity, and saccade amplitude might increase when there is more cognitive load on people [53]. Our results are consistent with the findings that the saccade numbers increased significantly, and the means of saccade velocity amplitude also increased. In addition, the workers’ mean and standard deviation of pupil diameter increased averagely, which is consistent with the previous finding that pupil diameter generally increases with an increase in mental load [29].

### 4.2. Performance of Prediction Models

Machine learning methods have been widely used in past studies for cognition prediction among construction workers. This study applied machine learning methods to measure the workers’ mental workload caused by heat exposure. The results show that the model applying eight HRV features could obtain the highest accuracy (90.00%) in classifying the mental workload in the two thermal conditions. Using the HRV features achieved a higher accuracy than using the eye movement features (78.33%) and the combined features of HRV and eye movement (88.33%). The results exemplify the advantages of HRV characteristics in measuring the mental workload due to heat exposure.

Additionally, the results show that selecting the appropriate number of features is also crucial for the prediction results. As shown in Figure 4, the accuracy follows a trend of first increasing, then decreasing, and then increasing again as the number of features increases. This might be related to the correlation between features. On one hand, when more features are added, if there are highly correlated features, redundant information may be introduced, leading to overfitting of the model, which causes a decrease in accuracy; on the other hand, as more unrelated but distinguishable features are added, the model may once again capture useful information, and the accuracy increases.

Compared to the existing studies in the construction field, this study proposed a physiological approach using HRV features to measure workers’ mental workload due to heat exposure. The previous studies focused on the measurement of workers’ mental workload caused by task properties without the consideration of the workplace thermal conditions [18]. In addition, previous studies were limited to the application of EEG [54], while the EEG signals would be easily disturbed and pose many restrictions on workers. Regarding the application of HRV among the extant literature in the construction field, they focused on the measurement of physical fatigue [55,56]. HRV features showed excellent performance in detecting physical fatigue, with a prediction accuracy of up to 93.5% [57]. Our study examined the performance of HRV features in indicating workers’ mental workload. The findings of this study further revealed the potential of HRV features for construction occupational safety monitoring, which demonstrates that HRV applications in cognition predictive, i.e., mental workload, are also feasible.

More importantly, it is also advantageous to utilize the model applying HRV features in the field. On one hand, a single sensor is required to obtain the ECG signals, which reduces the difficulty of data collection in workplaces compared to using multiple sensors. On the other hand, the results show that the number of HRV features (n = 8) needed is relatively small. The small number means ECG data can be processed quickly, which is important for low-cost real-time monitoring. The high prediction accuracy (90.00%), good evaluation performance, and field realizability all recommend that the prediction model be a practical approach in job sites.

Supervised learning algorithms could utilize labeled training datasets to teach models to produce a desired output. Previous studies has shown that supervised machine learning is a promising method for performing cognitive states using HRV characteristics (e.g., [42,58]. Four algorithms were used in this study; KNN showed superior performances in predicting mental workload when using HRV features. KNN was reported to be compatible with large-scale samples [55], which could be considered even when dealing with a huge sample size. What is more, KNN is robust to noisy data, and it is a simple algorithm demanding a low computational cost. These advantages make it a promising approach for cognitive state detection when considering its industrial application.

### 4.3. Implications

Compared to previous literature in the construction field, which was limited to measures of mental workload due to task attributes, this study explores what physiological data can respond to workers’ mental workload due to heat exposure. Specifically, this study revealed the relationships between HRV, eye movement characteristics, and mental workload. These relationships can serve as a basis for feature screening for developing prediction models estimating mental workload. The findings also reveal that HRV can be a good monitor of mental workload due to heat exposure, demonstrating the potential of HRV for construction safety monitoring.

For the practical, this study could provide a feasible way to monitor mental workload due to heat exposure in workers. The study developed predictive models for measuring mental workload using HRV features, eye movement features, and their combined features, respectively. The construction site can choose the measurements based on the form of physiological data available. Moreover, the acquisition of both types of physiological data in the field is feasible. HRV is derived from ECG signals, which can be measured by unobtrusive sensors, such as wristbands, armbands, and chest straps. Eye movement features are expected to be obtained by installing cameras in the field to capture the eye images of the workers. Both the physiological signals can be acquired in a way that causes less disruption to the workers, and the signals are minimally interfered by the noise in the field. The results also show that a small number of features are needed, which also facilitates real-time computation of data for real-time monitoring. Excessive cognitive load would impair workers’ safety, and this objective and real-time approach of measuring mental workload would ultimately improve occupational safety.

### 4.4. Limitations and Future Work

There are two limitations in this study. First, individual factors may also contribute to mental workload. The invited subjects in this study were less experienced workers, as inexperienced workers may be more susceptible to the negative effects of mental workload. For workers with a great deal of experience, their physiological parameters may not vary in the same way as the novices, which would lead to the fact that the model created in this study may not be applicable to the measurement of mental workload for the more experienced workers. The group of experienced workers is suggested to be further investigated in the future.

Secondly, this study was dedicated to the study of mental workload caused by heat exposure, and the experimental study chose a typical thermal condition for simulation (33 °C, 70% RH). However, if the thermal condition varies significantly from this study, e.g., an extremely hot environment (e.g., 38 °C or higher) or a slightly hot environment (e.g., around 30 °C), the physiological parameters of the workers may change differently and thus, the developed models would not be applicable. Therefore, it is recommended that future studies should consider different thermal conditions.

Third, the experiment did not include a multi-task complexity gradient under the same heat exposure conditions. As a result, the model cannot classify mental workload levels when the heat exposure condition remains the same. In future work, it is suggested to integrate subjective workload ratings and multi-task conditions to further refine the modeling of mental workload and improve the model’s ability to classify different levels of mental workload.

## 5. Conclusions

This study examines the performances of HRV and eye movement features in measuring construction workers’ mental workload caused by heat exposure. The results show that using eight HRV features through the KNN algorithm could achieve an accuracy of 90.00% in classifying mental workload in normothermic and hyperthermic conditions, with the evaluation performance: Recall = 0.933, Precision = 0.875, F1 = 0.903, AUC = 0.887. Applying four eye movement features through the SVM algorithm could achieve a prediction accuracy of 78.33%, with the evaluation performance: Recall = 0.800, Precision = 0.774, F1 = 0.787, AUC = 0.844. When using twenty-four both HRV and eye movement features through RF algorithm, the prediction accuracy can be up to 88.33%, with the evaluation performance: Recall = 0.867, Precision = 0.897, F1 = 0.881, AUC = 0.912. Monitoring mental workload is crucial for ensuring worker safety, and heat exposure is an important factor that triggers mental workload. This study achieved monitoring of mental workload using HRV and eye movement data, and the results could fill the gap that the issue of heat exposure-induced mental workload measurement was ignored. It is also promising to be applied in job sites to monitor mental workload because using both data collection in the field is feasible.

## Figures and Tables

**Table 1:** Descriptions of HRV features employed.

HRV Feature	Unit	Description (Equation)
Time-domain features		
mRR	[ms]	The mean of RR intervals ($\frac{\sum_{i=1}^{N}(RR_{i})}{N}$)
SDRR	[ms]	The standard deviation of RR intervals ($\sqrt{\frac{\sum_{i=1}^{N}{\left(RR_{i}-mRR\right)}^{2}}{N-1}}$)
RMSSD	[ms]	The square root of the mean squared differences between successive RR intervals ($\sqrt{mean{\left(RR_{i+1}-RR_{i}\right)}^{2}}$)
PNN50	[%]	Number of interval differences in successive RR intervals greater than 50 ms ($\frac{count{\left(\left|RR_{i+1}-RR_{i}\right|\right)\;}_{>\;50\;\mathrm{ms}}}{N-1}\times 100\%$)
Frequency-domain features		
VLF	[ms^2^]	Absolute powers of very low frequency band (0–0.04 Hz)
LF	[ms^2^]	Absolute powers of low frequency band (0.04–0.15 Hz)
HF	[ms^2^]	Absolute powers of high frequency band (0.15–0.4 Hz)
TP	[ms^2^]	The total energy of RR intervals
pVLF	[%]	Relative powers of VLF (VLF [ms^2^]/TP [ms^2^] × 100%)
pLF	[%]	Relative powers of LF (LF [ms^2^]/TP [ms^2^] × 100%)
pHF	[%]	Relative powers of HF (HF [ms^2^]/TP [ms^2^] × 100%)
LF/HF	[n.u.]	The ratio between LF and HF band powers
nLF	[n.u.]	Normalized low frequency power
nHF	[n.u.]	Normalized high frequency power
Non-linear features		
SD2/SD1	-	Ratio between SD2 and SD1
ApEn	-	Approximate entropy
SampEn	-	Sample entropy

**Table 2:** Descriptions of eye movement features employed.

HRV Feature	Unit	Description (Equation)
Fixation-based features		
Total fixation duration (TFD)	[s]	The whole period of fixation time during the whole task
Average fixation duration (MFD)	[s]	The ratio of total fixation periods to the total number of fixations
Fixation count (TFC)	/	The whole number of fixations during the whole task
Saccade-based features		
Saccade count (SC)	/	The number of saccade events during the task
Average velocity of saccades (MSV)	[degree/s]	The average angular velocity of all saccade events
Average amplitude of saccades (MSA)	[degree]	The average amplitude of all saccades
Pupil features		
Mean pupil diameter (MPD)	[mm]	The values of mean pupil diameter during the whole task
Standard deviation of pupil diameter (SPD)	[mm]	The standard deviation of pupil diameter during the whole task

**Table 3:** Paired T-test results of HRV features.

HRV Features	NT-Task (Mean ± SD)	HT-Task	t	*p*
		(Mean ± SD)		
mRR	799.47 ± 77.66	794.46 ± 82.38	0.716	0.480
SDRR	24.25 ± 7.91	26.97 ± 6.38	−1.929	0.064
RMSSD	27.47 ± 10.78	28.29 ± 9.30	−0.659	0.515
PNN50	9.09 ± 1.80	9.26 ± 1.45	−0.121	0.904
VLF	45.32 ± 11.14	26.55 ± 2.88	2.093	0.045
LF	205.04 ± 85.83	212.34 ± 189.43	0.195	0.847
HF	260.16 ± 147.62	234.10 ± 121.13	1.346	0.189
TP	510.52 ± 202.67	472.99 ± 223.92	1.521	0.139
pVLF	0.081 ± 0.082	0.058 ± 0.025	1.711	0.097
pLF	0.436 ± 0.167	0.421 ± 0.221	0.332	0.742
pHF	0.482 ± 0.185	0.521 ± 0.211	−0.864	0.394
LF/HF	1.407 ± 1.55	1.456 ± 2.275	−0.098	0.922
nLF	44.62 ± 20.88	48.34 ± 13.81	−1.417	0.167
nHF	54.92 ± 21.41	51.07 ± 14.51	1.483	0.149
SD2/SD1	1.54 ± 0.47	1.73 ± 0.65	−2.073	0.047
ApEn	0.97 ± 0.18	0.99 ± 0.15	−1.366	0.183
SampEn	1.76 ± 0.22	1.88 ± 0.15	−3.371	0.002

**Table 4:** Paired T-test results of eye movement features.

Eye Movement Features	NT-Task (Mean ± SD)	HT-Task	t	*p*
		(Mean ± SD)		
Total fixation duration (TFD)	19.69 ± 3.26	22.63 ± 6.88	−2.266	0.031
Average fixation duration (MFD)	9.79 ± 3.90	5.99 ± 2.74	5.161	<0.001
Total fixation count (TFC)	2381.33 ± 1222.07	4240.33 ± 1739.35	−6.533	<0.001
Saccade count (SC)	2217.33 ± 1151.18	3597.33 ± 1618.87	−5.472	<0.001
Average velocity of saccades (MSV)	552.79 ± 74.17	561.00 ± 74.94	−0.555	0.583
Average amplitudes of saccades (MSA)	16.36 ± 2.79	16.57 ± 2.82	−0.466	0.645
Mean pupil diameter (MPD)	3.319 ± 0.584	3.349 ± 0.441	−0.370	0.714
Standard deviation of pupil diameter (SPD)	0.550 ± 0.180	0.630 ± 0.272	−1.395	0.174

**Table 5:** Evaluation results of prediction models with the highest accuracy.

Applied Features	Prediction Accuracy	Model Evaluations
HRV features (n = 6)	KNN, 90.00%	Recall = 0.900, Precision = 0.900, F1 = 0.900, AUC = 0.905
HRV features (n = 8)	KNN, 90.00%	Recall = 0.933, Precision = 0.875, F1 = 0.903, AUC = 0.887
Eye movement features (n = 8)	KNN, 78.33%	Recall = 0.800, Precision = 0.774, F1 = 0.787, AUC = 0.771
Eye movement features (n = 5)	KNN, 78.33%	Recall = 0.800, Precision = 0.774, F1 = 0.787, AUC = 0.779
Eye movement features (n = 4)	KNN, 78.33%	Recall = 0.800, Precision = 0.774, F1 = 0.787, AUC = 0.817
Eye movement features (n = 4)	SVM, 78.33%	Recall = 0.800, Precision = 0.774, F1 = 0.787, AUC = 0.844
Eye movement features (n = 3)	KNN, 78.33%	Recall = 0.800, Precision = 0.774, F1 = 0.787, AUC = 0.783
Eye movement features (n = 3)	LDA, 78.33%	Recall = 0.800, Precision = 0.774, F1 = 0.787, AUC = 0.790
Eye movement features (n = 3)	SVM, 78.33%	Recall = 0.800, Precision = 0.774, F1 = 0.787, AUC = 0.836
HRV and eye movement features (n = 24)	RF, 88.30%	Recall = 0.867, Precision = 0.897, F1 = 0.881, AUC = 0.912

## Data Availability

The raw data supporting the conclusions of this article will be made available by the authors on request.
